# Socioeconomic differences in animal food consumption: Education rather than income makes a difference

**DOI:** 10.3389/fnut.2022.993379

**Published:** 2022-11-03

**Authors:** Urte Klink, Jutta Mata, Roland Frank, Benjamin Schüz

**Affiliations:** ^1^Institute for Public Health and Nursing Research, Prevention and Health Promotion, University of Bremen, Bremen, Germany; ^2^Health Psychology, Department of Social Sciences, University of Mannheim, Mannheim, Germany; ^3^GfK Verein, Nürnberg, Germany

**Keywords:** socioeconomic status, eating behavior, diet, sustainable diet, animal foods

## Abstract

**Background/aims:**

Evidence points toward more sustainable and health-conscious dietary behaviors among individuals with higher socioeconomic status. However, these differences vary considerably depending on which indicator of socioeconomic status is examined. Here, we present a systematic parallel investigation of multiple indicators of socioeconomic status as predictors of animal food consumption frequency and selected food-related behaviors in Germany.

**Methods:**

Data from the German subsample of two large representative European consumer studies (Study 1 *n* = 1,954; Study 2 *n* = 2,045) was used. We assessed the associations between the socioeconomic indicators income, current occupation as well as education and consumption frequency of animal foods and selected food-related behaviors in separate ordinal logistic regressions.

**Results:**

Individuals with higher educational attainment engaged in more sustainable and health-conscious dietary behaviors, indicated by significant associations between educational attainment and the consumption frequency of animal foods. Low- and middle-income participants consumed processed meat more frequently (Study 1 only; medium income: OR 1.5, CI 1.09–2.05, *p* = 0.012; low income: OR 1.43, CI 1.01–2.05, *p* = 0.047) and fish less frequently (Study 2 only; medium income: OR 0.76, CI 0.59–0.97, *p* = 0.026; low income: OR 0.061, CI 0.46–0.82, *p* < 0.001) than participants with high income. Current occupation did not predict the consumption of animal foods or food-related behaviors. Intake frequency of animal-based foods indicates that most participants exceeded national dietary recommendations for meat and processed meat and remained below recommendations for fish and dairy/eggs intake.

**Conclusion:**

Educational attainment appears to be the strongest and most consistent socioeconomic indicator of sustainable dietary choices in Germany based on current large, representative studies. Future efforts should be directed toward education interventions about nutrition and interpretation of food labels to compensate for differences in dietary behavior among groups with different levels of education.

## Introduction

Socioeconomic disparities in health status have widely been reported. Socioeconomically disadvantaged groups experience higher mortality and morbidity rates for coronary heart disease, type 2 diabetes and some cancers and are more likely to be overweight and obese (1, 2). Among the risk factors for such non-communicable diseases, diet is particularly important (3). A mostly plant-based diet complemented with minimal amounts of animal-based foods is considered to be a diet beneficial to both people and the planet (4). Individuals from less advantaged backgrounds are more likely to have healthier and more sustainable eating behaviors, including a higher intake of fruit and vegetable and a lower intake of animal foods (5, 6). Poor diet quality and lower adherence to dietary recommendations in socioeconomically disadvantaged groups likely contribute to their poorer health status (5–7). To better understand reasons for such differences in diet, it is crucial to examine differences in consumption patterns among groups with various indicators of social disadvantage.

The relationship between socioeconomic indicators and diet has been examined on the basis of dietary patterns, food and nutrient intake (6). While the evidence points to healthier and more sustainable dietary behaviors among those with higher socioeconomic status, results regarding the predictive power of individual socioeconomic indicators for animal food intake varies substantially, and socioeconomic differences in Germany have rarely been examined.

Consumption of different food groups affect health outcomes to varying degrees. While regular consumption of plant-based food groups like fruits and vegetables are commonly known to offer health-protective benefits and should therefore be encouraged, consumption of other foods has become known to increase the risk for various diseases and all-cause mortality (8, 9). Here, in particular, the consumption of animal-based foods is relevant, as an increased intake of cholesterol, fat and saturated fat through animal products is linked to various diseases such as dyslipidaemia (10). The consumption of red and processed meat is linked to an increased risk for cancer, especially colorectal cancer (11), and even small reductions in the consumption of red and processed meats substantially reduce the carbon footprint in food production (12). Current recommendations in Germany suggest limiting intake of red meats to no more than 300–600 g per week (this equals e.g. 2–4 hamburger patties/week), consuming sea fish once or twice a week due to their high content of health-promoting omega-3 fatty acids and iodine, daily consumption of milk and dairy products due to their calcium content, and limiting egg consumption to three eggs a week (13). Consumption of food of animal origin, and meat in particular, above recommended amounts has detrimental effects on the environment due to the high greenhouse-gas emissions and a reduction in consumption has been called for to curb the looming climate crisis (4, 14). However, the associations between socioeconomic indicators and consuming animal-based foods are very heterogenous, owing in particular to different operationalizations of socioeconomic status. Socioeconomic status describes an individual's standing within society based on the distribution of tangible and intangible resources along dimensions such as income, occupation and education (15, 16). Each measure has its strengths and limitations, and implies different ways of affecting dietary behaviors (17). Importantly, their assessment in population-based studies is not consistent, and there is a need to identify a uniform approach to this and determine socioeconomic predictors of specific health behaviors. Regarding education level, Méjean et al. (18) identified significant inverse associations with red meat, processed meat and poultry intake, but not for other animal food groups. Touvier et al. (19) found compliance with seafood recommendations to be associated with higher educational level but no significant associations were found for meeting the meat/seafood/egg recommendation and education level. More fish consumers could be found in the high-income compared to the low-income group (18), and others have also found a positive relationship between income and fish intake (20). Varying results have been found regarding egg consumption: one study found no differences in egg consumption and education (21), while another has found a significant inverse association between egg consumption and education level (22). In their review, Darmon and Drewnoski (6) identified higher egg intake among individuals of lower socioeconomic status. Sanchez-Villegas et al. (23) found higher cheese consumption in participants with higher educational levels but no associations between milk consumption and education in their systematic review of European studies.

In addition to the intake of selected food groups, dietary behavioral patterns also have health implications and are likely to differ according to socioeconomic indicators. In the current analyses, we examine food label reading as well as the consumption of regional foods, frozen fruits and vegetables, prepared fruits and vegetables, convenience foods, and dietary supplements. Among these, food label use has been associated with improved dietary patterns (24–26). The use of regional or local foods could indicate a preference for quality and traditional foods, as these have been perceived as having higher quality than foods produced further away (27). The use of frozen fruits and vegetables, prepared fruits and vegetables, and convenience foods may indicate a preference for easy-to-prepare meals that require less time and effort. The regular use of convenience foods may also indicate low cooking skills as preparation requires little effort (28). Further, because convenience foods often have low nutritional value (29), their frequent intake may indicate an unhealthy eating pattern. Taking dietary supplements has been associated with adopting other healthy habits, such as eating a healthy diet, regular exercise, and tobacco avoidance (30). To date, there are only few studies examining socioeconomic differences in these dietary behavioral patterns and have mostly found associations with education: those with higher educational attainment were more likely to read food labels (25) and to use supplements (30) but less likely to use convenience foods than those with lower educational attainment (28).

In sum, given the scarcity of the literature, there is a need to systematically identify and compare different socioeconomic predictors of specific food consumption and other food-related health behaviors. Specifically, we are not aware of studies that have focused on animal groups in the German population.

Therefore, the aim of the current studies is to evaluate the predictive power of the most commonly used socioeconomic indicators for the consumption frequency of animal food groups and selected food-related behaviors. For this purpose, data from the German subsample of two large representative European consumer studies are examined.

## Methods

### Study sample and procedure

This study is a secondary analysis of previously published anonymized survey data from the non-profit branch of a market research organization (31); as such, the IRB of the University of Mannheim informed that no ethics approval was required. Data assessment was conducted in agreement with the ethical guidelines of European market research companies. The data are not publicly available.

Subsamples from two large European consumer studies conducted in 2011 (Study 1) and 2017/18 (Study 2) were used for this investigation. Data for both studies were collected and prepared by GfK Consumer Insight (Gesellschaft für Konsumforschung) on behalf of GfK Verein, a non-profit organization for market research from Nuremberg, Germany. GfK Verein is now NIM (Nuremberg Institute for Market Decisions).

Study 1 originally consisted of 10,226 individuals from eight European countries (Austria, France, Germany, Italy, the Netherlands, Poland, Spain and the U.K.) and Russia. Data were collected as part of the Food and Nutrition Study in 2011. More details are provided by Mata et al. (32).

Study 2 originally consisted of 10,134 individuals from six European countries (Germany, France, Spain, Poland, Italy, the U.K.), Russia and the USA. Data was collected as part of the Consumer Study 2018 conducted by GfK Consumer Insight on behalf of the non-profit organization for market research, GfK Verein, in winter of 2017/18.

In order to present the data of the consumer studies in a clear and appropriate manner/scope and since no comparable studies have been carried out in the German population, only data of German participants from both consumer studies were used for the present analyses. In the original 2011 study (Study 1), 2,062 respondents from Germany were included. Participants were excluded if they had replied “Don't know” to questions regarding food-related behaviors (*n* = 108), resulting in a sample size of 1,954 participants. For Study 2, 2,045 study participants from Germany were included.

In both studies, sampling was realized using the quota procedure, a non-probability sampling method that creates a sample that includes individuals who are representative of a population with respect to specific characteristics. In the current study participants were representative of the population of Germany with respect to gender, age, employment status/occupation, household size, and region of residence.

In quota sampling, a population is stratified into mutually exclusive sub-groups. Interviewers were told to find a certain number of individuals to match a sub-group previously determined. The interviews were carried out as computer-assisted personal interviews in the respective language of each country with the exception of the USA, where assessments took place online, and Russia, where paper-and-pencil were used.

### Indicators of socioeconomic status

Three indicators of socioeconomic status were used for the analysis: level of education, current occupation, and personal income level. Education was categorized as being low, medium, and high based on International Standard Classification of Education (ISCED) categories and corresponded to years of education. Accordingly, low-level education translates to not yet having graduated from school or having received basic education with or without completing an apprenticeship. In Germany, basic education requires 9 years of schooling. Medium-level education translates to a mid-level education without the qualification for university entrance, which can be achieved after a minimum of 10 years of schooling. High-level education represents a higher-level education with qualification for university entry or having graduated from university or college. To reach qualification for university entry, 12–13 years of schooling are required. Participants were categorized into low, middle, and high net income if they earned <1,000, 1,000–1,999, or ≥2,000 €/month, respectively. This categorization was based on the Organization of Economic Co-operation and Development (OECD) equivalence scale for income. Current occupation was assigned according to participants' responses regarding their type of employment (e.g., type of blue-collar work) and working status (employed, unemployed, retired, in school) following GfK categories.

### Dietary behaviors

In Study 1 only, participants answered survey questions regarding food-related behaviors. For the present analysis, six topics were deemed of interest. Regarding food labels, participants were asked how often they read ingredient lists on food packaging, and a four-point Likert scale was used as response option. The survey included statements regarding how often certain food products were used. Participants were asked how often they use regionally produced foods, frozen fruits and vegetables, prepared foods such as ready-made salads, convenience foods such as canned soups and whether they take nutritional supplements often. Participants were asked on a four-point Likert scale to what extent these applied to them.

In Study 1 and 2, participants were asked to classify how frequently they consumed four different animal food groups on a seven-point Likert scale: meat (pork, beef, veal, lamb, game, poultry), sausage and ham (processed meat), fish and seafood (e.g. crabs, prawns, mussels, oysters), and dairy products and eggs (milk, cottage cheese, yogurt, cheese).

Participants were classified as being either non-vegetarian, moderate vegetarian, ovo-lacto-vegetarian or vegan according to their frequency of animal food consumption. Vegans do not consume any animal foods. Ovo-lacto-vegetarians avoid meat, processed meat and fish but eat dairy products and eggs. Moderate vegetarians eat one of these food groups once a week: meat or processed meat or fish and seafood. The other two foods groups are eaten less than once a week.

### Statistical analysis

All analyses were conducted using R version 4.0.2 and the R-packages *MASS* version 7.3–55 (33) and *table1* version 1.4.2 (34). Prior to performing the statistical analysis, several socioeconomic measures were tested for multicollinearity to select independent predictors, specified by a variance inflation factor of below 5. These measures included personal net income, household income, level of education, current occupation (profession), and working status. Personal net income, level of education and current occupation (profession) were chosen. As dependent variables were scaled ordinally, ordinal logistic regression was used to determine socioeconomic predictors associated with food label reading, intake of food products and frequency of consumption of animal-based foods. Additionally, age and sex were controlled for as confounders in the logistic regression models.

## Results

### Study participants

Characteristics of both study populations are presented in Table 1. Both populations were similar in age (47.7 vs. 48.9 years), gender distribution (51.8 vs. 51.7% female), education, and current occupation. However, differences between study populations can be observed in their income level. In Study 2, relatively more participants fell into the high-income group than in Study 1 (12.8 vs. 17.6%). In addition, sex-specific income differences between and within the study populations can be observed.

**Table 1:** Sample characteristics for 2011 and 2017/18, overall and by sex.

	**2011**			**2017/18**		
	**Total** ***n* = 1,954**	**Male** ***n* = 941**	**Female** ***n* = 1,013**	**Total** ***n* = 2,045**	**Male** ***n* = 987**	**Female** ***n* = 1,058**
**Education level** ***n*** **(%)**
High	377 (19.3)	197 (20.9)	180 (17.8)	490 (24.0)	254 (25.7)	236 (22.3)
Medium	758 (38.8)	340 (36.1)	418 (41.3)	813 (39.8)	362 (36.7)	451 (42.6)
Low	749 (38.3)	374 (39.7)	375 (30.7)	665 (32.5)	321 (32.5)	344 (32.5)
Don't know	70 (3.6)	30 (3.2)	40 (3.9)	77 (3.8)	50 (5.1)	27 (2.6)
**Income level** ***n*** **(%)**
High	251 (12.8)	193 (20.5)	58 (5.7)	360 (17.6)	262 (26.5)	98 (9.3)
Medium	696 (35.6)	378 (40.2)	318 (31.4)	697 (34.1)	307 (31.1)	390 (36.9)
Low	541 (27.7)	156 (16.6)	385 (38.0)	426 (20.8)	155 (15.7)	271 (25.6)
No answer	466 (23.8)	214 (22.7)	252 (24.9)	562 (27.5)	263 (26.6)	299 (28.3)
**Current occupation** ***n*** **(%)**
Manager	62 (3.2)	40 (4.3)	22 (2.2)	62 (3.0)	40 (4.1)	22 (2.1)
Self-employed	126 (6.4)	75 (8.0)	51 (5.0)	130 (6.4)	86 (8.7)	44 (4.2)
White-collar employee	585 (29.9)	217 (23.1)	368 (36.3)	666 (32.6)	238 (24.1)	428 (40.5)
Blue-collar worker	209 (10.7)	140 (14.9)	69 (6.8)	205 (10.0)	147 (14.9)	58 (5.5)
Currently not working - had been working before	677 (34.6)	322 (34.2)	355 (35.0)	652 (31.9)	299 (30.3)	353 (33.4)
Currently not working - had never been working	289 (14.8)	144 (15.3)	145 (14.3)	320 (15.6)	173 (17.5)	147 (13.9)
Don't know	6 (0.3)	3 (0.3)	3 (0.3)	10 (0.5)	4 (0.4)	6 (0.6)
**Age mean (SD)**	47.7 (18.2)	47.7 (18.6)	47.6 (17.8)	48.9 (19.3)	48.1 (19.9)	49.6 (18.6)
**Female** ***n*** **(%)**	1,012 (51.8)			1,058 (51.7)		

### Socioeconomic differences in food-related behaviors (Study 1)

Table 2 shows results of the ordinal logistic regression assessing the association between socioeconomic measures and six food-related behaviors.

**Table 2:** Odds ratios, 95% confidence intervals and *p*-values for various food-related behaviors 2011.

**Predictors**	**Food labels** ^**1**^			**Regional foods** ^**2**^			**Frozen fruits & vegetables** ^**3**^			**Prepared fruits & vegetables** ^**4**^			**Convenience foods** ^**5**^			**Supplements** ^**6**^		
	***Odds ratios***	***CI***	***p*-Values**	***Odds ratios***	***CI***	***p*-Values**	***Odds ratios***	***CI***	***p*-Values**	***Odds ratios***	***CI***	***p*-Values**	***Odds ratios***	***CI***	***p*-Values**	***Odds ratios***	***CI***	***p*-Values**
**Education level**																		
High	1.00	(referent)		1.00	(referent)		1.00	(referent)		1.00	(referent)		1.00	(referent)		1.00	(referent)	
Medium	0.55	0.44–0.70	**<0.001**	0.90	0.71–1.15	0.408	1.21	0.96–1.53	0.104	1.17	0.93–1.49	0.183	1.28	1.01–1.62	**0.045**	0.92	0.72–1.16	0.482
Low	0.34	0.26–0.44	**<0.001**	0.76	0.59–0.99	**0.043**	1.19	0.92–1.52	0.186	1.13	0.87–1.45	0.356	1.43	1.10–1.85	**0.007**	0.87	0.68–1.12	0.290
Don't know	0.43	0.26–0.73	**0.002**	1.23	0.72–2.10	0.450	0.72	0.43–1.23	0.236	0.86	0.51–1.45	0.569	0.83	0.49–1.41	0.490	0.60	0.35–1.03	0.068
**Income level**																		
High	1.00	(referent)		1.00	(referent)		1.00	(referent)		1.00	(referent)		1.00	(referent)		1.00	(referent)	
Medium	0.77	0.58–1.03	0.075	1.05	0.78–1.40	0.751	0.97	0.74–1.28	0.840	1.05	0.79–1.39	0.749	1.38	1.04–1.83	**0.026**	0.81	0.61–1.08	0.154
Low	1.03	0.75–1.42	0.833	1.20	0.86–1.67	0.276	0.92	0.67–1.26	0.593	0.79	0.57–1.09	0.147	1.26	0.92–1.74	0.151	0.62	0.45–0.86	**0.004**
No answer	0.84	0.62–1.13	0.246	1.36	1.00–1.87	0.054	0.96	0.71–1.29	0.790	0.70	0.52–0.95	**0.021**	1.09	0.80–1.47	0.596	0.77	0.57–1.05	0.098
**Current occupation**																		
Manager	1.00	(referent)		1.00	(referent)		1.00	(referent)		1.00	(referent)		1.00	(referent)		1.00	(referent)	
Self-employed	0.71	0.39–1.26	0.238	1.11	0.62–1.98	0.737	0.71	0.41–1.23	0.219	0.66	0.37–1.16	0.145	0.83	0.47–1.48	0.532	1.11	0.63–1.96	0.724
White-collar employee	0.73	0.44–1.21	0.224	0.87	0.52–1.44	0.582	0.64	0.39–1.03	0.069	0.74	0.45–1.22	0.241	1.04	0.62–1.73	0.887	1.21	0.74–2.00	0.456
Blue-collar worker	0.58	0.33–1.02	0.057	0.83	0.47–1.46	0.517	0.88	0.51–1.51	0.653	0.74	0.43–1.28	0.285	1.12	0.64–1.96	0.702	0.99	0.57–1.73	0.966
Currently not working - had been working before	0.68	0.40–1.14	0.141	0.77	0.46–1.31	0.337	0.51	0.31–0.83	**0.007**	0.62	0.37–1.03	0.063	0.89	0.53–1.51	0.666	1.22	0.73–2.04	0.447
Currently not working - had never been working	0.40	0.23–0.69	**0.001**	0.67	0.39–1.17	0.163	0.54	0.32–0.92	**0.023**	0.68	0.40–1.17	0.162	1.03	0.59–1.79	0.920	1.47	0.86–2.53	0.164
Don't know	0.73	0.18–3.03	0.664	2.91	0.64–14.09	0.170	1.51	0.37–6.26	0.566	0.52	0.10–2.66	0.424	1.47	0.34–6.31	0.603	2.62	0.62–11.00	0.182
**Age** ^**†**^	1.01	1.00–1.02	**0.001**	1.04	1.03–1.05	**<0.001**	1.00	0.99–1.00	0.123	0.98	0.97–0.98	**<0.001**	0.96	0.96–0.97	**<0.001**	1.01	1.01–1.02	**<0.001**
**Sex**																		
Male	1.00	(referent)		1.00	(referent)		1.00	(referent)		1.00	(referent)		1.00	(referent)		1.00	(referent)	
Female	2.56	2.14–3.07	**<0.001**	1.43	1.19–1.71	**<0.001**	1.33	1.11–1.59	**0.002**	0.73	0.61–0.87	**<0.001**	0.66	0.55–0.79	**<0.001**	1.33	1.11–1.59	**0.002**
Observations **1,954**																		

^1^“Normally, the manufacturers of the food you buy list the ingredients on the label. How often do you read these labels?” Possible answers on a 4-point Likert scale: Just about never, never/Seldom/Frequently/Very frequently, almost always.

^2^“I often use food items produced or grown in the region where I live.” *

^3^“I often use frozen vegetables and frozen fruits.” *

^4^“I frequently buy and/or eat prepared foods such as ready-made salads, pre-cut vegetables etc.” *

^5^“I often use convenience foods, canned soup etc.” *

^6^“I often take vitamin and mineral supplements.” *

^*^Possible answers on a 4-point-Likert-scale: Does not apply at all/Applies to a lesser degree/Applies to a certain degree/Applies fully and completely.

^†^Continuous variable.

Bold *p*-values indicate statistically significant association.

In general, current occupation was not significantly associated with food-related behaviors. However, participants currently not working used frozen fruits and vegetables less frequently than participants in management positions.

Compared to highly educated participants, those with medium and low education were less likely to read food labels but were more likely to use convenience foods.

Income level was only significantly associated with consumption frequency of convenience foods and usage of supplements. Compared to high-level income participants, middle-income participants were 38% more likely to use convenience foods, while low-income participants were 38% less likely to use nutritional supplements.

Additionally, sex differences could be observed. Women were more likely to use food labels, regional foods and frozen fruits and vegetables, and take supplements but were less likely to use convenience foods and prepared fruits and vegetables than men.

### Socioeconomic differences in consumption frequency of animal products

Most participants in both studies regularly consumed animal products (96.47 and 91.49% for Study 1 and 2, respectively). Only small groups of participants classified as moderate vegetarian (3.17 and 7.87%), ovo-lacto-vegetarian (0.36 and 0.34%) or vegan (0 and 0.29%). Tables 3, 4 show results of the ordinal logistic regression assessing the association between socioeconomic indicators and consumption frequency of four animal food groups. Compared to higher education, having low or medium education was associated with higher consumption of meat and processed meat and lower consumption of fish/seafood as well as dairy/eggs.

**Table 3:** Odds ratios, 95% confidence intervals and *p*-values for frequency of consumption of animal products 2011.

**Predictors**	Meat*			Processed meat*			Fish and seafood*			Dairy products and eggs*		
	***Odds ratios***	***CI***	***p*-Values**	***Odds ratios***	***CI***	***p*-Values**	***Odds ratios***	***CI***	***p*-Values**	***Odds ratios***	***CI***	***p*-Values**
**Education level**												
High	1.00	(referent)		1.00	(referent)		1.00	(referent)		1.00	(referent)	
Medium	1.48	1.13–1.96	**0.005**	1.66	1.27–2.16	**<0.001**	0.60	0.48–0.75	**<0.001**	0.86	0.67–1.12	0.265
Low	1.47	1.09–1.97	**0.011**	1.37	1.03–1.81	**0.028**	0.49	0.39–0.63	**<0.001**	0.64	0.49–0.84	**0.001**
Don't know	2.14	1.15–4.00	**0.017**	1.34	0.73–2.44	0.340	0.67	0.40–1.11	0.121	0.99	0.56–1.77	0.983
**Income level**												
High	1.00	(referent)		1.00	(referent)		1.00	(referent)		1.00	(referent)	
Medium	1.05	0.75–1.48	0.769	1.50	1.09–2.05	**0.012**	0.88	0.67–1.16	0.364	0.89	0.66–1.21	0.463
Low	1.13	0.77–1.66	0.526	1.43	1.01–2.05	**0.047**	0.95	0.70–1.29	0.747	1.05	0.75–1.48	0.769
No answer	1.02	0.71–1.47	0.907	1.30	0.93–1.82	0.123	0.92	0.69–1.23	0.575	1.20	0.87–1.67	0.263
**Current occupation**												
Manager	1.00	(referent)		1.00	(referent)		1.00	(referent)		1.00	(referent)	
Self-employed	0.84	0.42–1.66	0.617	0.62	0.33–1.15	0.129	1.11	0.64–1.91	0.715	0.62	0.33–1.14	0.124
White-collar employee	0.68	0.37–1.22	0.201	0.63	0.37–1.08	0.093	1.16	0.71–1.87	0.555	0.58	0.33–1.01	0.056
Blue-collar worker	0.77	0.40–1.49	0.446	1.03	0.57–1.87	0.925	0.93	0.55–1.58	0.792	0.40	0.22–0.73	**0.003**
Currently not working - had been working before	0.75	0.41–1.37	0.357	0.82	0.47–1.44	0.488	1.05	0.64–1.73	0.837	0.58	0.32–1.01	0.055
Currently not working - had never been working	0.66	0.34–1.24	0.196	0.59	0.33–1.06	0.079	1.00	0.60–1.69	0.987	0.81	0.44–1.45	0.476
Don't know	0.51	0.08–3.43	0.479	0.31	0.05–1.87	0.204	0.60	0.14–2.43	0.482	0.37	0.06–2.52	0.299
**Age** ^**†**^	0.99	0.99–1.00	0.080	1.00	0.99–1.01	0.773	1.02	1.01–1.03	**<0.001**	1.01	1.00–1.01	**0.006**
**Sex**												
Male	1.00	(referent)		1.00	(referent)		1.00	(referent)		1.00	(referent)	
Female	0.37	0.30–0.46	**<0.001**	0.38	0.31–0.46	**<0.001**	1.08	0.91–1.28	0.389	2.02	1.68–2.45	**<0.001**
Observations **1,954**												

*From questionnaire: “How often do you consume these foods…?” Possible answers on a 7-point-Likert-scale: Just about never/Less frequently/Once a month/Several times a month/Once a week/Several times a week/On a daily basis.

^†^Continuous variable.

Bold *p*-values indicate statistically significant association.

**Table 4:** Odds ratios, 95% confidence intervals and *p*-values for frequency of consumption of animal products 2017/18.

**Predictors**	Meat*			Processed meat*			Fish and seafood*			Dairy products and eggs*		
	***Odds ratios***	***CI***	***p*-Values**	***Odds ratios***	***CI***	***p*-Values**	***Odds ratios***	***CI***	***p*-Values**	***Odds ratios***	***CI***	***p*-Values**
**Education level**												
High	1.00	(referent)		1.00	(referent)		1.00	(referent)		1.00	(referent)	
Medium	1.41	1.12–1.78	**0.004**	1.58	1.25–2.00	**<0.001**	0.64	0.51–0.78	**<0.001**	0.79	0.63–1.00	**0.047**
Low	1.91	1.47–2.49	**<0.001**	1.27	0.98–1.66	0.075	0.58	0.46–0.73	**<0.001**	0.72	0.56–0.93	**0.013**
Don't know	1.58	0.93–2.69	0.092	1.32	0.78–2.25	0.296	0.77	0.47–1.24	0.284	1.28	0.76–2.17	0.346
**Income level**												
High	1.00	(referent)		1.00	(referent)		1.00	(referent)		1.00	(referent)	
Medium	1.09	0.83–1.45	0.534	1.08	0.82–1.42	0.575	0.76	0.59–0.97	**0.026**	0.89	0.68–1.16	0.384
Low	1.10	0.78–1.53	0.594	1.18	0.85–1.65	0.327	0.61	0.46–0.82	**0.001**	1.14	0.82–1.58	0.428
No answer	0.89	0.67–1.20	0.453	0.85	0.63–1.13	0.257	0.76	0.59–0.98	**0.033**	0.56	0.42–0.74	**<0.001**
**Current occupation**												
Manager	1.00	(referent)		1.00	(referent)		1.00	(referent)		1.00	(referent)	
Self-employed	0.84	0.44–1.57	0.590	0.90	0.49–1.64	0.723	1.20	0.70–2.06	0.505	1.11	0.61–2.04	0.734
White-collar employee	0.81	0.46–1.40	0.459	0.84	0.50–1.43	0.528	0.83	0.52–1.33	0.435	0.96	0.56–1.64	0.875
Blue-collar worker	0.93	0.50–1.71	0.815	1.37	0.76–2.45	0.290	0.68	0.41–1.15	0.155	0.93	0.52–1.68	0.818
Currently not working - had been working before	0.67	0.37–1.18	0.168	0.99	0.57–1.71	0.970	0.80	0.49–1.31	0.383	1.02	0.59–1.78	0.942
Currently not working - had never been working	0.63	0.34–1.14	0.135	0.76	0.43–1.35	0.354	0.68	0.41–1.14	0.140	0.71	0.40–1.28	0.254
Don't know	0.34	0.09–1.32	0.107	0.22	0.06–0.80	**0.018**	2.22	0.68–7.47	0.189	0.44	0.11–1.74	0.242
**Age** ^**†**^	1.00	0.99–1.01	0.893	1.01	1.00–1.01	**0.012**	1.01	1.00–1.01	**0.004**	1.00	1.00–1.01	0.396
**Sex**												
Male	1.00	(referent)		1.00	(referent)		1.00	(referent)		1.00	(referent)	
Female	0.51	0.42–0.61	**<0.001**	0.58	0.48–0.70	**<0.001**	1.42	1.20–1.67	**<0.001**	2.03	1.69–2.44	**<0.001**
Observations **2,045**												

*From questionnaire: “How often do you consume these foods…?” Possible answers on a 7-point-Likert-scale: Just about never/Less frequently/Once a month/Several times a month/Once a week/Several times a week/On a daily basis.

^†^Continuous variable.

Bold *p*-values indicate statistically significant association.

Income affected fish/seafood consumption in Study 2 only—participants with medium and low incomes consumed fish significantly less frequently than those with high income. Additionally, in Study 1 only, participants with medium and low incomes were significantly more likely to frequently consume processed meat than participants with high income.

Current occupation did not influence the consumption of foods of animal origin. In general, women consumed fish/seafood, dairy/eggs more regularly, and less meat and processed meat than men.

### Changes in consumption frequency of animal products

**Figure 1:**
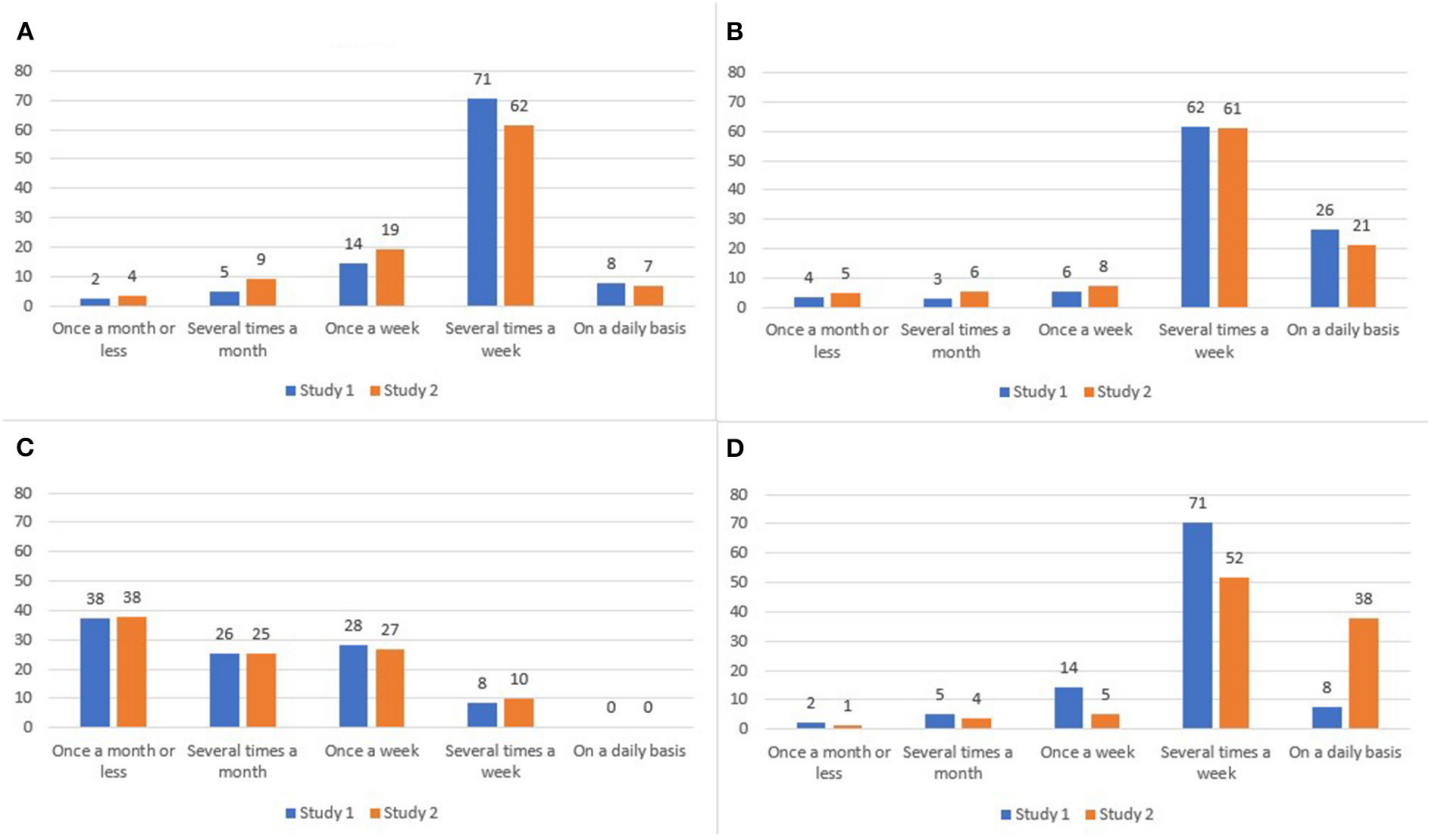
Responses (%) regarding consumption frequency of **(A)** meat, **(B)** processed meat, **(C)** fish/seafood, and **(D)** dairy/eggs for Study 1 (2011) and Study 2 (2017/18).

Some changes in the consumption frequency of animal foods between 2011 and 2017/18 could be observed. Compared to Study 1 (2011), participants in Study 2 (2017/18) consumed meat and processed meat less frequently (i.e., 79% consumed meat several times a week or on a daily basis in 2011, 69% in 2017/18; 88% processed meat in 2011 vs. 82% in 2017/18). However, meat and processed meat continued to be eaten regularly. Consumption of dairy/eggs appeared to have increased, while fish/seafood consumption showed no change. Most participants consumed fish/seafood once a week or less, while dairy/eggs were consumed several times a week or daily (Figure 1).

## Discussion

This study examined indicators of socioeconomic status as predictors for animal food consumption and selected food-related behaviors. We found significant associations between participants' educational background and the consumption frequency of animal food groups, suggesting more sustainable and health-conscious dietary habits among those with high education. Participants of low and middle income consumed processed meat more frequently than participants of high income, but this association was only significant in Study 1. Likewise, low- and middle-income participants consumed fish/seafood less frequently than high-income participants, but this association was only significant in Study 2. Current occupation did not predict food-related behaviors and animal food consumption frequency in either study. A large proportion of participants exceeded current dietary recommendations for meat and processed meat and did not meet recommendations for fish/seafood or dairy/eggs, thereby also failing to adhere to sustainable dietary guidelines (4). Despite meat and fish being comparably expensive foods, education was the strongest and most consistent predictor of consumption, not income. Given that available information on food products, such as food labels, was also more frequently used by people with higher education, this suggests that education – including about low-threshold readily available information such as food labels – might be a promising step toward healthier nutrition.

### Education

In both study populations, those with low and medium levels of education were more likely to regularly consume meat and processed meat than participants with a high level of education. Several authors have previously reported an inverse relationship between education and meat and processed meat intake (18, 19, 35–38). In both studies, participants with low and mid-level education were less likely to frequently consume fish/seafood than participants with a high level of education, which is consistent with the results in other populations (17, 39, 40).

Likewise, participants with low and mid-level education consumed dairy/eggs less frequently than participants with high education. In their systematic review, Sanchez-Villegas et al. (23) found higher cheese consumption in participants with higher educational levels but no associations between milk consumption and education. Varying results have been found regarding egg consumption: one study reported no differences in egg consumption by education (21), while another has found more egg purchasing in lower education (22).

Convenience foods were more frequently used by participants with low and mid-level education than those with high education. In agreement with our results, education has previously been inversely associated with convenience food consumption (28, 41). Convenience foods are often regarded as processed foods and may encompass a variety of foods, including ready-made meals, fast foods, snack foods, and packaged/canned/frozen/pre-prepared foods. They are considered the least healthy and among the most unsustainable of dietary options due to their low nutritional value and high energy input during production (29). McGowan et al. (42) found that participants with lower cooking and food skills had no or few educational qualifications, which could ultimately influence convenience food consumption as these require little cooking skills.

Education is considered the most consistent and reliable indicator of SES as it depicts processes that occur early in life and tend to remain stable over time or illness (15). The relationship between education and diet is thought to reflect a person's capability to access, construe and likely apply health-related information (16, 17, 43). Therefore, higher education suggests greater knowledge about diet and health, resulting in food decisions that align with current dietary recommendations. Indeed, higher educational level has been associated with better diet quality (44–46) and higher nutritional knowledge, as demonstrated in several studies (47–49). Some authors have found nutrition knowledge to mediate the relationship between education or socioeconomic status and diet quality (46, 48, 50).

Additionally, education level is one of the factors influencing food label reading, as shown in a conceptual framework of consumers' understanding and use of information on food labels (51). In qualitative studies, participants of low socioeconomic status have indicated that sufficient nutrition literacy was needed to use nutrition information labels (52), and difficulties reading food labels may act as a barrier to making informed food-purchasing decisions (53). Satia et al. (25) found healthful behavior characteristics and psychosocial factors, such as moderate physical activity and healthful eating self-efficacy, to be significantly associated with nutrition label reading, indicating their use as part of a cluster of health-promoting behaviors. Indeed, reading and understanding food labels may be another mechanism that connects educational attainment and dietary behaviors, as suggested in previous investigations (24–26).

### Income

Generally, in our studies, the relationships between meat and processed meat consumption frequency with income were less strong than with education, indicating income to have a comparatively smaller effect on meat consumption. However, a large proportion of participants in both populations (24% in Study 1, 28% in Study 2) did not report their current income, which may have affected the reliability of our results in this regard. Simultaneously, income can be rather unstable over time and may thus have a smaller effect on health behaviors. It has previously been described as an indicator of current living standards (54).

One investigation in the US (55) and one in Germany (56) failed to find an association between meat consumption and income, however, two more recent German studies found an inverse relationship (36, 57). This association has further been confirmed on a global level by Vranken et al. (58), while they also highlighted that low-income groups tend to prefer cheaper cuts of meat.

Furthermore, in low-income groups, low concern about the health risks of frequent meat consumption (and low fruit and vegetable consumption) has been reported (59), again indicating a lower knowledge of the diet-health relationship in lower socioeconomic groups. However, it has also been argued that not necessarily knowledge gaps, but, amongst others, cost is perceived as the main barrier to healthy food access (60, 61). Low-income groups widely view fruits and vegetables as beneficial to health but also expensive (61, 62), thus likely siding with more energy-dense foods, like processed meat or convenience foods.

Cost may also have contributed to the positive relationship between fish/seafood consumption frequency and income level, which was consistent with other findings (39, 40). One of the most commonly perceived barriers to fish consumption is its high price, which is considered higher than other protein sources such as meat (63, 64). It is unclear whether the significant association in Study 2 is due to price increases for fish and seafood as other animal foods have seen similar price increases (65) or whether other mechanisms were responsible.

### Current occupation

Several studies have previously identified differences in dietary intake across occupational groups; however, results appear somewhat inconclusive and may vary by gender (17, 23, 66).

It has been suggested that education determines occupational class and hence income. The effect of education on income is mainly mediated through occupation, as has been highlighted by Galobardes et al. (17) and Lahelma et al. (67). Thus, individuals with a higher level of education tend to have higher-status jobs and consequently, greater financial resources due to higher income. However, more than income and education, occupation is a measure of social prestige. It is believed to influence dietary behavior partly *via* workplace cultures and social relationships (17). Work can also influence health behaviors through physical demands, compensation and benefits, and exposure to hazards (68). Generally, however, employment or occupational status is assessed variably in epidemiological research, leading to a number of problems. Firstly, respondents are often either grouped by employment status (e.g., employed, unemployed, retired) or by level of skills or knowledge required for their job (occupational categories, e.g. unskilled workers, skilled workers, lower or upper non-manuals, etc.), making it difficult to compare results between studies. Secondly, assigning respondents to these groups inadvertently creates rather heterogeneous groups that often do not reflect similar workplace environments or social networks, masking possible health effects. Thirdly, work rankings are relatively unstable over time, as new demands and economic needs can shift rankings in terms of income and status (15).

While some authors have found associations between occupation and health behaviors or outcomes (19, 44, 45) and different occupational classification schemes have been investigated for their effectiveness (69), meaningful and reliable assessment of occupation remains a challenge.

### Gender differences

Overall, women showed more health-promoting and sustainable dietary behaviors. They consumed meat and processed meat less frequently, and fish/seafood as well as dairy/eggs more frequently than male participants. Furthermore, women read food labels more often, consumed regional foods and frozen fruits and vegetables, took dietary supplements more frequently, and consumed prepared fruits and vegetables and convenience foods less regularly than men. Our findings are in line with previous research. For instance, women consume less meat (35, 36), are more likely to be vegetarian (36, 38) and have a higher fruit and vegetable intake than men (70). Women also have higher nutrition knowledge (46), higher beliefs in the importance of healthy eating (71) and placed more value into sustainable diet practices compared to men (72).

### Changes in the consumption frequency of animal products

We observed some changes in the consumption frequency of animal products between the study periods 2011 and 2017/18. Participants in 2017/18 consumed meat and processed meat less frequently than those in 2011. These findings align with official data from German government agencies, showing that the average meat consumption has slightly declined over the last decade (73). In 2020, Germans consumed 57.3 kg of meat per person, a reduction from 62.8 kg in 2011 (73, 74). Meat still holds a high status in Germany society (37), but whether the reduction is due to an increased awareness of health, animal welfare and environmental concerns related to frequent meat consumption still needs to be investigated, specifically in the German population.

Additionally, the 2017/18 study population consumed dairy/eggs more frequently than the 2011 population. According to official German governmental data, consumption of dairy products has been declining for the past two decades, with a reduction of 3.3 kg of milk and cheese per person per year between 2011 and 2017/18 (75). However, egg consumption has been increasing in the same time frame (76). Unfortunately, dairy and egg consumption frequency was not recorded separately; thus, it is not possible to determine whether the increase in consumption in our sample was due to an increase in egg or dairy consumption, or both.

### Strengths and limitations

Strengths of this investigation include the large sample size representative of the German population. We used two samples with data collected at different points in time, and both samples were comparable in age and gender distribution. The 7-year interval provides the opportunity to observe potential behavior changes. We used the three most important and most commonly assessed socioeconomic measures to investigate their predictive potential on dietary behaviors. Furthermore, we examined a variety of diet-related behaviors with potential health implications.

Limitations include the dietary intake assessment. Consumption frequency does not provide information on the quantities consumed, which would have required a different dietary assessment method. Dairy and egg consumption frequency was summed up in one variable, and therefore differences by socioeconomic status could not be detected. Under general practice in nutritional epidemiology, they should have been assessed separately because they are different food groups and their consumption is recommended in varying amounts. A large number of participants in both populations (24% in Study 1, 28% in Study 2) did not report their current income, which may have affected the reliability of our results. Additionally, prior categorization of the socioeconomic indicators may have hindered their predictive power, particularly for income and current occupation. While the categorization for income led to an even distribution of the groups, a different classification may have led to a stronger effect. The categorization of current occupation likely led to heterogenous groups. Future studies could benefit from applying a more reliable measurement tool assessing work prestige.

We found that most participants (>90%) frequently consumed animal products, but <1% classified as vegetarian or vegan. This finding contradicts results of the DEGS1 study whereby 4.3% of the German population follow a vegetarian diet (38), suggesting that vegetarian and vegan participants were underrepresented, at least in the 2017/18 study population. This could indicate that our data was not representative of the German population in this respect.

Furthermore, the GfK Verein has not examined the consumption of animal foods and food-related behaviors in other consumer studies, meaning that further investigations such as changes over time were not possible.

## Conclusion

The present study offers insight into which indicators of socioeconomic status have the most predictive power for animal food consumption and select food-related behaviors. Educational achievement appears to be the best predictor for animal food consumption and food label reading but had less predictive power for other food-related behaviors. Income was only predictive for consuming processed meat and fish/seafood.

Future efforts should be directed toward comprehensive and practical nutrition education interventions, for example about nutrition and interpretation of food labels, to compensate for differences in dietary behavior among groups with different levels of education. On a population level, even small changes in individual consumption of red and processed meats can have substantial health and environmental benefits (12). Further research should be conducted to improve understanding of the relationship between dietary habits, occupational status, and income.

## Author's note

GfK Verein is now called Nuremberg Institute for Market Decisions (NIM).

## Data availability statement

The raw data supporting the conclusions of this article will be made available by the authors, without undue reservation.

## Ethics statement

Ethical review and approval was not required for the study on human participants in accordance with the local legislation and institutional requirements. Written informed consent to participate in this study was provided by the participants' legal guardian/next of kin.

## Author contributions

UK conceived of the study, analyzed data, and wrote the original draft manuscript. JM supported study conception and data interpretation and revised the manuscript. RF conceptualized and coordinated data collection and revised the manuscript. BS conceived of the study, planned data analysis and interpretation, and revised the manuscript. All authors contributed to the article and approved the submitted version.

## Conflict of interest

The data were collected when RF was employed by the GfK Verein, a non-profit branch of the Gesellschaft für Konsumforschung. The remaining authors declare that the research was conducted in the absence of any commercial or financial relationships that could be construed as a potential conflict of interest.

## Publisher's note

All claims expressed in this article are solely those of the authors and do not necessarily represent those of their affiliated organizations, or those of the publisher, the editors and the reviewers. Any product that may be evaluated in this article, or claim that may be made by its manufacturer, is not guaranteed or endorsed by the publisher.
